# Research on the Construction and Application of Breast Cancer-Specific Database System Based on Full Data Lifecycle

**DOI:** 10.3389/fpubh.2021.712827

**Published:** 2021-07-12

**Authors:** Yin Jin, Wang Junren, Jiang Jingwen, Sun Yajing, Chen Xi, Qin Ke

**Affiliations:** ^1^School of Computer Science and Engineering, University of Electronic Science and Technology of China, Chengdu, China; ^2^West China Biomedical Big Data Center, West China Hospital, Sichuan University, Chengdu, China; ^3^Medical Big Data Center, Sichuan University, Chengdu, China; ^4^Chengdu Zhixin Electronic Technology Co., Ltd, Chengdu, China

**Keywords:** breast cancer, disease-specific database, metadata, data governance, data security sharing, knowledge graph

## Abstract

Relying on the Biomedical Big Data Center of West China Hospital, this paper makes an in-depth research on the construction method and application of breast cancer-specific database system based on full data lifecycle, including the establishment of data standards, data fusion and governance, multi-modal knowledge graph, data security sharing and value application of breast cancer-specific database. The research was developed by establishing the breast cancer master data and metadata standards, then collecting, mapping and governing the structured and unstructured clinical data, and parsing and processing the electronic medical records with NLP natural language processing method or other applicable methods, as well as constructing the breast cancer-specific database system to support the application of data in clinical practices, scientific research, and teaching in hospitals, giving full play to the value of medical big data of the Biomedical Big Data Center of West China Hospital.

## Introduction

With the rapid development of new technologies such as big data and artificial intelligence, the medicine overlaps with such disciplines as information technology, computer science, and cyber security in more and more aspects. Particularly, thanks to the constant advancement of medical technology, the process for screening, diagnosis and treatment of diseases is being expanded to generate various new data. Based on different data modalities, artificial intelligence technology has been widely applied in the field of medicine (1–5). This research focuses on breast cancer which results in the second highest cancer mortality in women (6) and its screening, diagnosis and treatment strategies which have developed from single surgical therapy to a comprehensive treatment mode that combines surgical therapy, chemotherapy, radiotherapy, endocrinotherapy, and targeted therapy, forming a multi-disciplinary team (MDT) of breast cancer. Multi-source heterogeneous data, such as electronic medical record data, image data and gene data, were generated in the whole diagnosis and treatment process to drive the disease diagnosis and treatment and disease research into a big data era of disease-specific research (7).

Currently, the most fundamental challenge confronting the medical research institutions in the development of disease-specific data research is how to integrate multi-source heterogeneous data and build a disease-specific database, so as to support the discovery of potential diagnosis and treatment knowledge patterns from the massive medical data. For example, the data are too much to be screened manually by clinicians; the attributes of the same data element are described in different ways across hospitals or even across information systems of the same hospital, e.g., the same drug may be identified by different codes. As unstructured data such as electronic medical records contain medical information of important value, the difficulty lies in the effective and accurate extraction of such information. Based on the medical big data governance activities in China, Li et al. proposed a big data governance framework for medical data in China through literature review, expert consulting and structural modeling, providing an important reference for data governance framework in this research (8). This paper aims to integrate two heterogeneous clinical data sources, i.e., unstructured medical records and structured clinical data, through clinical text analysis and knowledge extraction; to break the information barriers within the organization and between clinical departments and to promote data sharing among medical centers in combination with patient information from multiple clinical data sources; to establish the disease-specific data standards in accordance with international industry standards and then to construct a multi-modal knowledge graph specific to breast cancer; finally to build a disease-specific database system for the purpose of analyzing disease characteristics, thus providing supports in clinical decision-making and rational drug use to clinicians in the diagnosis and treatment of breast cancer.

## The Involvement of Breast Cancer Patients

Patients pathologically diagnosed with breast cancer are prospectively registered in the Breast Cancer-specific Database System at West China Hospital, Sichuan University since 2008 (9, 10). Medical records, diagnostic pathology reports, treatment records are recorded by oncologists. All patients are followed by outpatient visit or telephone at 3–4-month intervals within 3 years after diagnosis, 6-month intervals within 4–5 years, and then annually. The characteristics description of breast cancer patients included in the database is shown in Table 1.

**Table 1:** Characteristics description among breast cancer patients at recruitment.

	**Group**	**Value**
Age, median (IQR)		41.0 (47.0, 55.0)
Sex	Female	7697 (99.6%)
BMI, median (IQR)		20.83 (22.86, 24.97)
Menopause status, No (%)	Yes	2999 (38.8%)
	No	4693 (60.7%)
	Unknown	38 (0.5%)
Stage, No (%)	0	0
	I	1619 (25%)
	II	3427 (33.5%)
	III	1927 (29.8%)
	Unknown	757 (11.8%)
pT status, No (%)	0	308 (4.0%)
	1	2532 (32.8%)
	2	3633 (47.0%)
	3	336 (4.3%)
	4	440 (5.7%)
	Unknown	481 (6.2%)
pN status, No (%)	0	3748 (48.5%)
	1	2227 (28.9%)
	2	856 (11.1%)
	3	783 (10.1%)
	Unknown	116 (1.4%)
ER	Negative–	2323 (30.1%)
	Positive+	5094 (65.9%)
	Unknown	313 (4.0%)
PR	Negative–	2680 (34.7%)
	Positive+	4737 (61.3%)
	Unknown	313 (4.0%)
HER2	Negative–	4555 (58.9%)
	Positive+	1856 (24.0%)
	Unknown	1319 (17.1%)
Ki67	<14%	1370 (17.8%)
	≥14%	5752 (74.4%)
	Unknown	608 (7.8%)

*ER, estrogen receptor; PR, progesterone receptor; HER2, human epidermal growth factor receptor-2; Ki-67, marker of proliferation Ki-67*.

## Overall Design Scheme for Breast Cancer-Specific Database System

**Figure 1:**
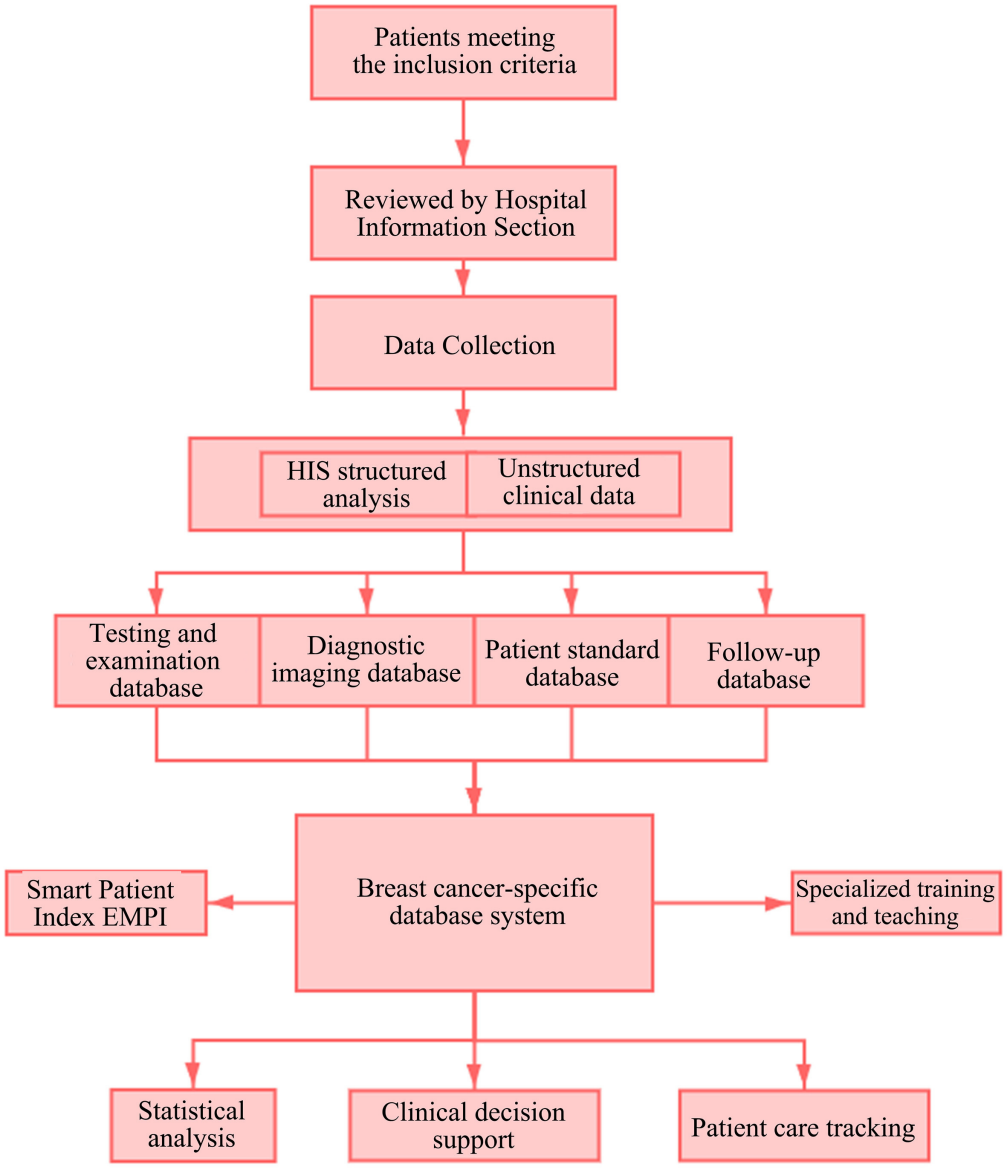
Overall design of breast cancer-specific database system. HIS, Hospital Information System; EMPI, Enterprise Master Patient Index.

Overall design thought of the breast cancer-specific database system is shown in Figure 1.

Several important oncology patient data systems of China and foreign countries (e.g., cancer registration software CanReg designed and developed by the Descriptive Epidemiology Unit of IARC\the cancer screening database of Chinese Anti-Cancer Association) were referred in the overall design and construction of the breast cancer-specific database system (11, 12). The database is composed of four parts: a patient standard database, a breast cancer malignancy-specific database, a diagnostic imaging database and a breast cancer patient follow-up visit database. Data governance is based on these four parts. The construction of the disease-specific database system involves governance, extraction and application. Governance is performed firstly to collect the current disease-specific data assets of the West China Hospital and sort out their meanings, ownership, etc. The next step is to conduct data classification and quality control to ensure the accuracy of data processing. The last step is to provide a unified standard interface services based on the governed and integrated disease-specific data center. In addition, main considerations in design of the disease-specific database include the application of the standardized and governed data in clinical diagnosis and treatment assistance and scientific research. The patient's medical record data can be viewed as a time series that captures the entire clinical process of collecting the patient's medical history, analyzing the condition, diagnosing, and treating the patient. Different data sources have different time spans, resulting in complex timing dependencies between events (13). Therefore, combined with the actual application scenarios and for better support to scientific research, the overall design and final data presentation of the disease-specific database are logically linked through the processes of admission registration, inpatient treatment, checkout and discharge, etc. in the chronological sequence, so as to establish a view for diagnosis and treatment based on full data lifecycle.

## Metadata-Centered Data Governance Scheme

### Standards–Establishment of Data Standards

#### Establishment of Dataset Standards

A regional breast cancer-specific database should include a complete range of datasets in the uniform format and meeting the normative standards. In addition, the database should incorporate the national and medical industry standards and all system datasets of medical institutions. A synonym database of the dataset names should be created. A standard database of breast cancer datasets should be created for the Center to provide a dataset graph for its application and corpus support for automatic identification of dataset names. The Center's database includes the scope, normative references, term abbreviations, dataset metadata attributes, and data element attributes. Standard information for each dataset includes the dataset name, identifier, classification, field description, definition, etc. The datasets are saved in four different databases based on data types, and subdivided into four modules and about 20 submodules. The oncology datasets and breast cancer-specific datasets are created accordingly with reference to different health industry standards. Some referenced dataset standards are shown in Table 2, and some collected fields of the breast cancer-specific dataset are shown in Table 3.

**Table 2:** Some referenced dataset standards.

**Classification**	**Standard name**
National health industry standards	Basic Dataset of Basic Information–Personal Information (WS 371-2012) Basic Items Data Set of Health Examination (T/CHIA 2-2018) Guidelines for Data Schema Description of Health Information (WS/T 304-2009) Classification and Coding for Value Domain of Health Data Element (WS364-2011)
International oncology specialized standards	SEER Program Coding and Staging Manual of National Cancer Institute (NCI) International Classification of Diseases, Tenth Revision (ICD-10) International Classification of Diseases for Oncology, Third Edition (ICD-O-3), etc.
Other standards	Supplement to current hospital datasets

*WS and WS/T refers to standards issued by Chinese Professional Committee on Health Information Standards; T/CHIA refers to standards issued by Chinese Health Information Association*.

**Table 3:** Some collected fields of the breast cancer-specific dataset.

**Classification**	**Field name**	**Database type**
Basic information	Patient ID, patient name, home address, contact number, first contact name, relationship with patient, ID number, gender, date of birth, height, weight, gender, age, marital status, occupation, ethnicity, ancestral home, nationality, etc.	Patient standard database
Inpatient information	Patient ID, time of medical records, reliability, medical unit admitted, nursing unit admitted, time of admission by the current medical unit, current medical unit, current nursing unit, date of diagnosis, time of admission, length of stay, time of discharge, transfer of departments, date of transfer from current medical unit, medical unit transferred, nursing unit transferred, attending physician, way of discharge, number of operations, etc.	Patient standard database
Progress note	Patient ID, chief complaint, clinical pathway ID, medical advice, observations, results of ward round, dosing regimen, summary opinions, etc.	Disease-specific database
Nursing assessment	Details of occupational exposure, smoking status, duration of smoking, average number of cigarettes, smoking cessation, duration of smoking cessation, drinking, duration of drinking, average number of drinks, allergy history, details of allergy history, diet, general health condition, vaccination history, past history of serious illness, details of serious illness, history of blood transfusion, trauma history, history of infectious diseases, details of infectious diseases, history of surgery, details of surgery, etc.	Patient standard database
Diagnostic information	Diagnosis category, diagnosis code, diagnosis name, pre- and post-operation diagnostic accordance, outpatient diagnostic accordance, clinical case diagnostic accordance, radiopathological diagnostic accordance, discrepancy between admission diagnosis and primary discharge diagnosis, cataloged diagnosis name splicing, cataloged diagnosis code splicing, first page diagnosis name splicing, tumor morphological code name, tumor morphological code, etc.	Disease-specific database
Physical examination	Body temperature, pulse rate, respiratory rate, blood pressure, general condition, skin mucosa, lymph nodes, head, hair distribution, eyes, ears, nose, mouth, face, neck, chest, lungs, heart, blood vessels, abdomen, genitalia, anorectum, spine and extremities, nervous system, routine examinations, specialist examinations, etc.	Patient standard database
Testing	LIS reported DR, test time, item number in test results, item name in test results, sample code, sample name, reference value range, quantitative result, item unit, label, result, etc.	Patient standard database
Examination	Mass size, distribution of lesions (single or multicenter), tumor location, presence of distant metastasis, etc.	Image database
Surgical anesthesia	Date, operation level, anesthesia level, incision type, anesthesiologist, operation code & name, operation time, surgeon, preoperative and postoperative diagnosis, preoperative chemotherapy, radiotherapy, anesthesia method, intraoperative bleeding, blood transfusion, etc.	Disease-specific database
Treatment information	Inpatient diagnosis and treatment plan, type of medical advice, item name of medical advice, frequency, usage, implementation date of medical advice, invalidation date of medical advice (for long-term treatment), source of medical advice, treatment means, etc.	Disease-specific database
Postoperative radiotherapy for tumor	Measurement of radiotherapy (single and cumulative), start and end time, adverse reactions, etc.	Disease-specific database
Postoperative chemotherapy for tumor	Chemotherapy regimen (i.e., drug type and dosage, route of administration), cycle, start and end time, adverse reactions, etc.	Disease-specific database
Disease progression and outcome	Conditions at admission, chief complaint, summary of medical record, course of disease (not available), discharge summary, main discharge diagnosis and treatment, etc.	Disease-specific database
Follow-up visit	Time, survival status, recurrence, metastasis, adverse reactions, etc.	Follow-up visit database
Charges	Charges for outpatient service, hospitalization, operation, examination, testing, drugs, etc.	Disease-specific database

*LIS, Laboratory Information Management System; DR, Digital radiography*.

#### Establishment of Data Element Standards

A local database of data element standards is established based on the national and industrial standards and in combination with the specific situation of the hospital. The local database includes data element indicators, normative references, term abbreviations, and data element directory. Data element standards specify the Chinese name, English name, identifier, definition, classification, data type, representation format, data threshold value, allowable value type and allowable value of data fields in the data dictionary, which are used to ensure the data quality. In the management of metadata, data elements may be classified and labeled, so as to establish a synonym database of the data elements. The local database describes the attributes of each data element, including Chinese field name, English field name, field name abbreviation, field type, field length, required or not, range or reference standards, notes and remarks. If there are any relevant international standards for the range of data elements, they can be referenced directly; otherwise, the range will be set by physicians and other professionals in combination with clinical experience. The set range standards will be saved together with other collected standards in the local database for the convenience of version management and subsequent updates. Table 4 shows partial attributes of some data elements.

**Table 4:** Partial attributes of some standard data elements.

**Field name**	**Field type**	**Field length**	**Required or not**	**Range or reference standard**
Gender code	varchar	10	Y	GB/T 2261.1-2003
Marital status code	varchar	10	Y	GB/T 2261.2-2003
Health insurance category code	varchar	10	N	CVO2.01.204 Table for Health Insurance Category Code
Medical history	varchar	200	N	0-No, 1-Yes
Registration category	varchar	20	Y	01-General clinic, 02-Emergency, 03-Specialty clinic, 04-Specialist clinic, 05-VIP clinic, 06-Disease-specific clinic, 09-Others
Diagnosis basis	varchar	20	Y	CT05.01.001
Prescription type/name	varchar	20	Y	01-General prescription, 02-Pediatric prescription, 03-Emergency prescription, 04-Narcotic drug prescription (Class I psychotropic drug prescription), 05-Narcotic drug prescription (Class II psychotropic drug prescription), 99-Others
Dosing frequency code	varchar	20	N	CV06.00.228
Examination site code	varchar	60	N	CV06.00.227
Surgical procedure code	varchar	20	Y	ICD-9-CM-3
Anesthesia mode code	varchar	20	Y	CV06.00.103
Surgical position code	varchar	10	Y	CV06.00.223
ASA physical status classification code	varchar	10	Y	CV05.10.021

*GB/T refers to standard issued by China National Institute of Standardization; CV and CT refer to classification code table in standard issued by China National Institute of Standardization*.

### Quality Control–Fusion and Governance of Multi-Source Heterogeneous Data

The structured medical data from HIS (Hospital Information System), LIS (Laboratory Information Management System), and follow-up visit system are integrated with the image data from PACS (Picture Archiving and Communication Systems). These data are acquired by building an ETL (Extract-Transform-Load) automation platform to perform incremental extraction at regular intervals on a daily basis, and complete data standardization and other processes during the extraction process. The unstructured data in the electronic medical record are structured through natural language processing and machine learning after data source access, and then saved in the disease-specific database.

**Figure 2:**
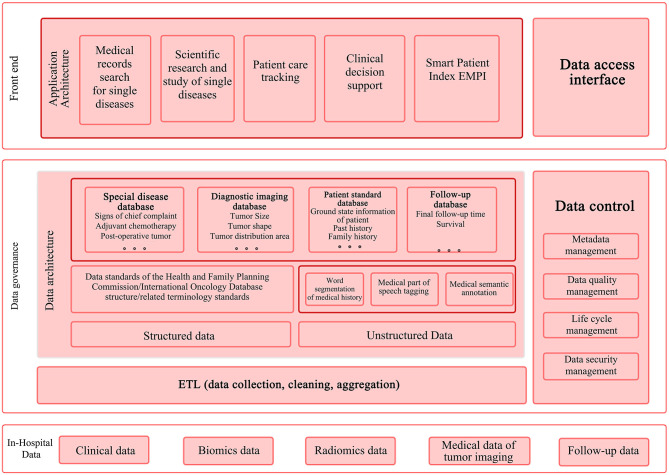
Data governance framework. ETL, Extract-Transform-Load.

Afterwards, the data in the four module databases are linked primarily based on the patient ID, thus breaking the information barriers within the organization and between clinical departments. Finally, the front-end application is supported by breast cancer-specific data for fully mining the data of full lifecycle about single disease and providing support for data analysis of multi-center joint scientific research projects. Specific processing methods are described below. The data governance framework is shown in Figure 2.

#### Structured Data Processing

**Figure 3:**
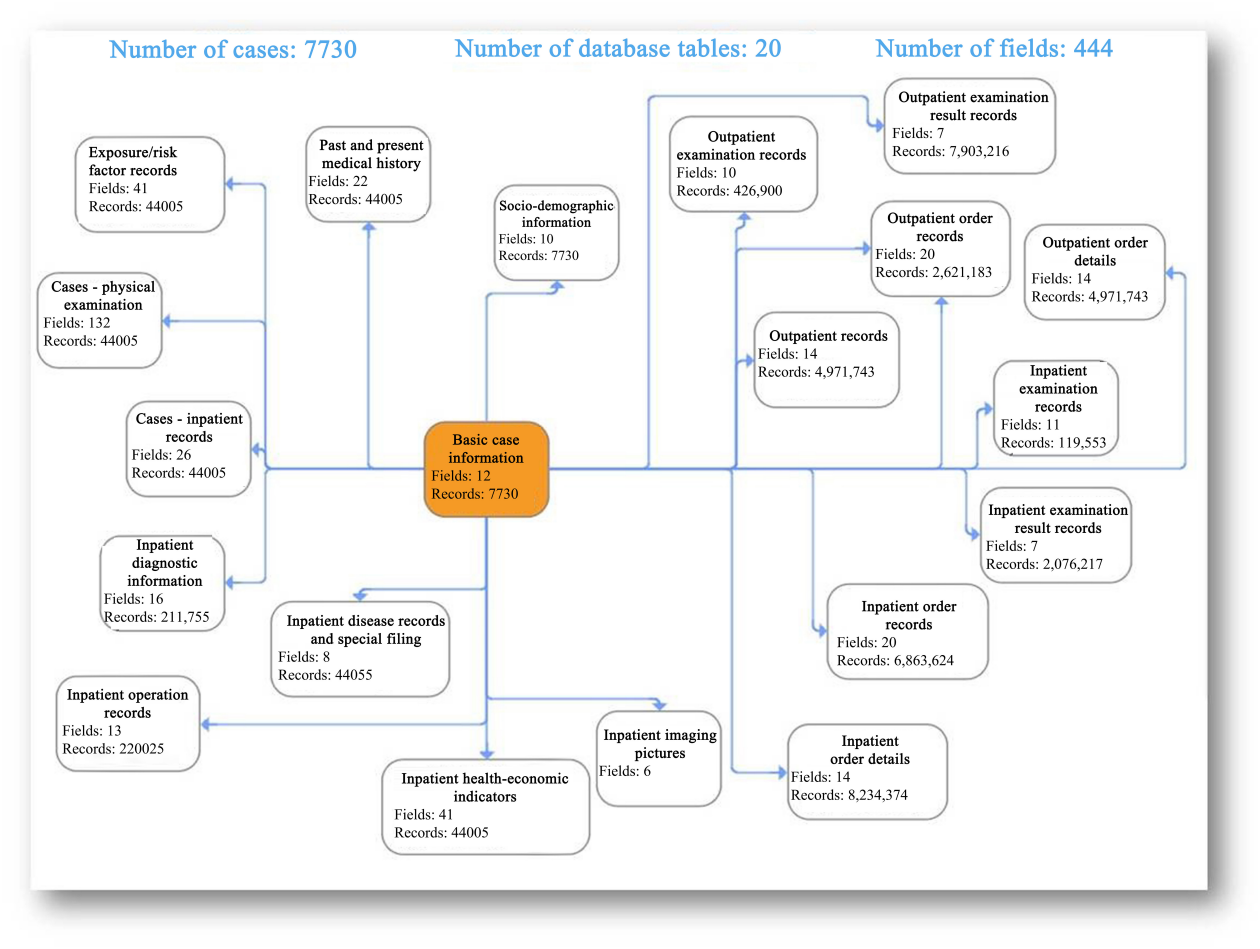
Database cleaning results.

Data acquisition (data reception or data capture) is performed through the data fusion platform for the data of breast cancer patients which are structured but exist in different systems. The source data are extracted, integrated and saved in the target database as per the following steps: (1) Establish a data source directory, and determine the connection mode, access permission, data storage directory, and interfaces of each data source; (2) Data cleaning and filtering: Establish data review rules, e.g., the gender can only be male, female, or unknown, the ID number can only be 18 digits, the patient ID cannot be blank, etc. Then, filter the data according to these rules, and save the unqualified data in a temporary database, with no need for data fusion. The cleaned data should not contain missing or incomplete data, repeated data and nonstandard data. See Figure 3 for the statistics of some cleaned data; (3) Map the original data in the data source database with the standard datasets in accordance with the specified data standards, and complete the range conversion of data elements at the same time to standardize the processing of breast cancer standard data, so as to complete the collection, and collation of multi-source data; (4) In the process of timed automatic incremental extraction of medical data, monitor the log for each extraction, and count the number of extraction records and completion for later failure rollback (14).

#### Unstructured Data Processing

**Figure 4:**
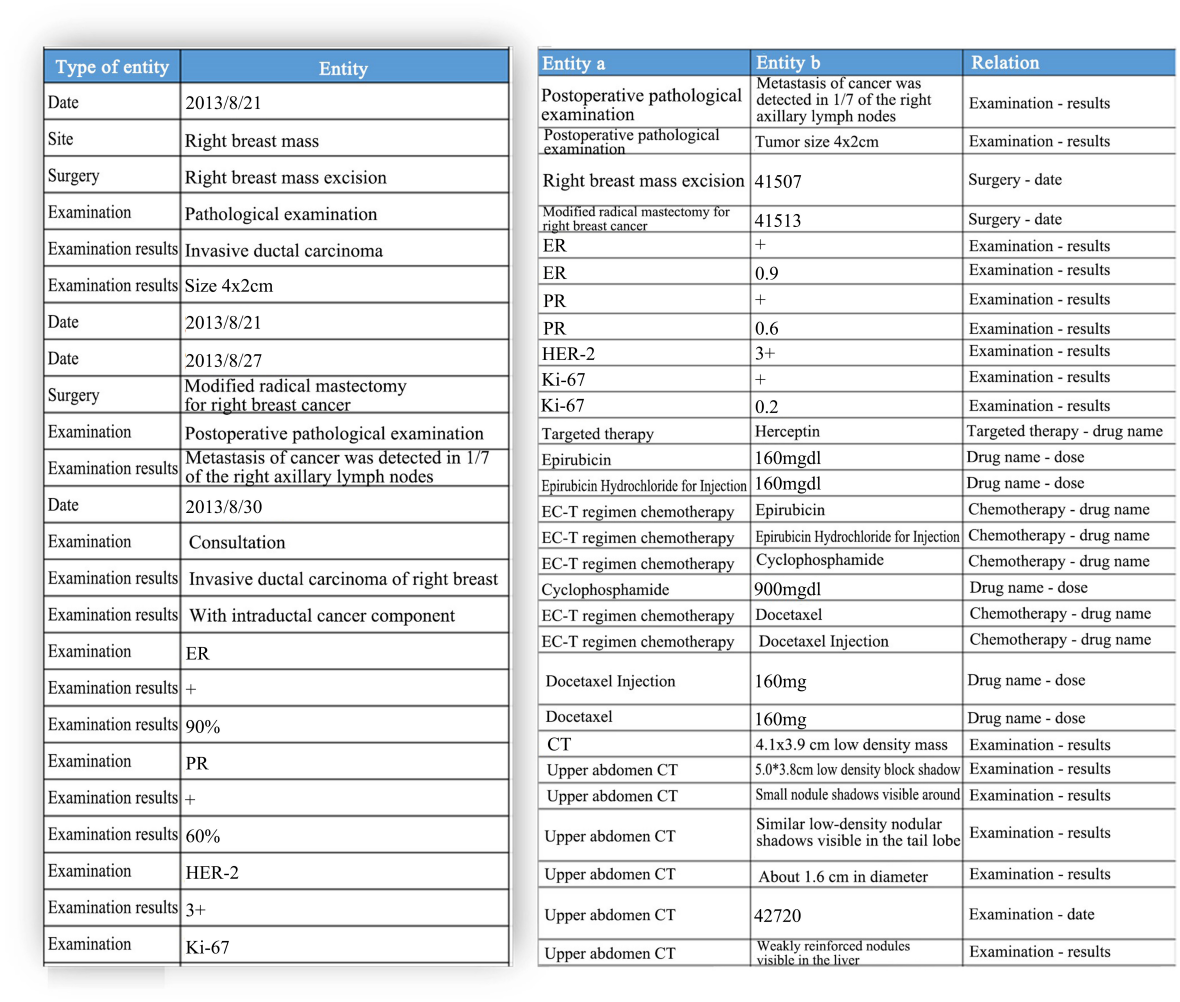
Parsing results of some electronic medical records. ER, estrogen receptor; PR, progesterone receptor; HER2, human epidermal growth factor receptor-2; Ki-67, marker of proliferation Ki-67.

Electronic medical records contain highly valuable medical data. The unstructured breast cancer data are parsed by the standard medical structure based on natural language text data, and the structured data correction, annotation and association tools are provided for clinicians to manage the annotation tasks (either by manual or automatic annotation) of the text data to be processed, so that the unstructured text data in breast cancer pathology reports and present medical history are transformed into analyzable structured data, providing a data basis for the construction of a subsequent consensus data link mining engine, an analysis tool for self-defined data link risk factors and a breast disease knowledge graph. Natural language texts (such as current medical history, color Doppler ultrasound description and pathological description) are annotated by professional physicians for entities and relations until 200 annotations, and preliminary training is developed, then back-annotation is performed using the trained model to assist the physicians in annotation of subsequent samples. At present, 1,000 samples have been annotated and trained, and completed for model training and model evaluation using NLP to make profound adjustment to model parameters. The model ability is evaluated, with the recognition accuracy of 80–85%, reaching the level of manual recognition by general physicians. The parsing results of some electronic medical records are shown in Figure 4.

#### Image Data Processing

**Figure 5:**
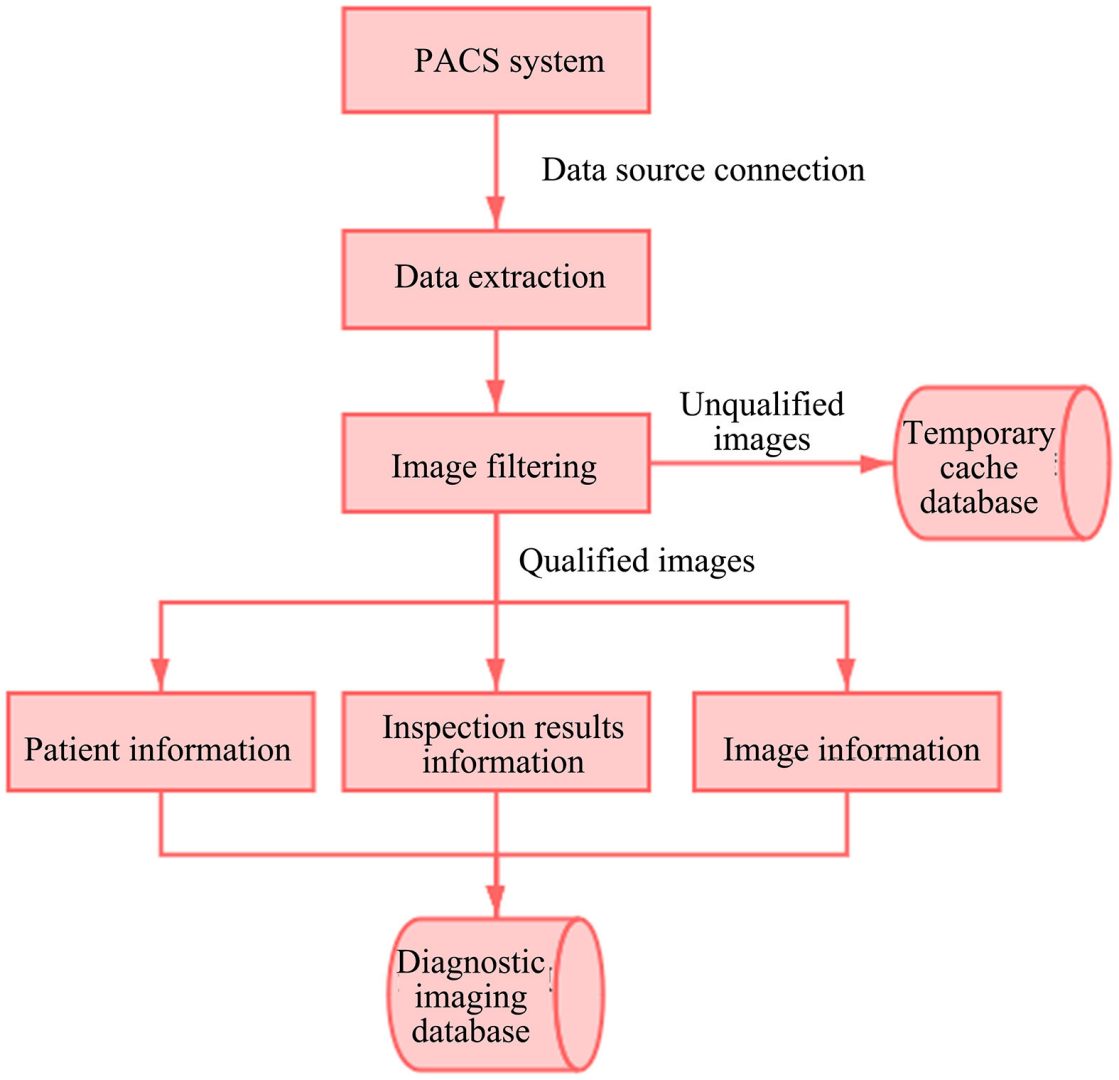
Image data governance process. PACS, Picture Archiving and Communication Systems.

Medical imaging technology has increasingly become an indispensable means for disease diagnosis, providing quick and accurate support for clinical practice. The imaging information about a patient's tumor site and the examination report issued by the radiologist are saved in the medical image data. In the process of image data governance, it is necessary to eliminate unqualified image data. With AI deep neural network machine learning technique, the machine can automatically distinguish the unqualified image data and annotate such data (15). The filtered unqualified images are saved in the temporary cache database and manually verified later. The qualified image data are extracted and saved together with basic patient information and report results in the diagnostic imaging database, and finally linked with the patient standard database and the disease-specific database based on the patient ID to connect the whole treatment process. The image data governance process is shown in Figure 5.

### Model-Building - Multi-Modal Breast Cancer-Specific Knowledge Graph

**Figure 6:**
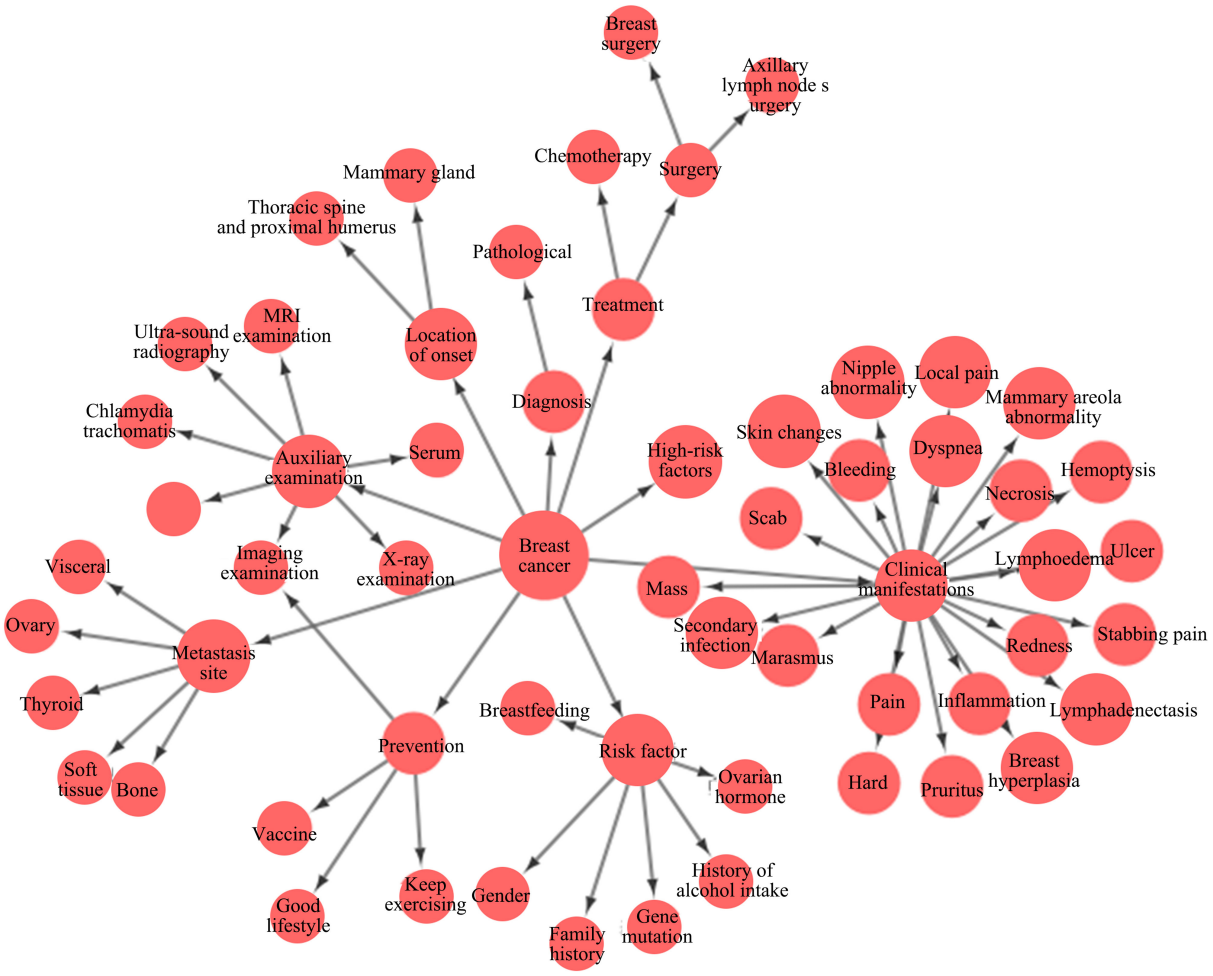
Partial breast cancer knowledge graph.

On the basis of the breast cancer-specific database system, a multi-modal breast cancer-specific knowledge graph is constructed to integrate texts, medical images, and even videos, voices and other rich media information, and to reflect the hierarchical relation among the entities and relations related to breast cancer such as pathogenesis, symptom characteristics, complications, treatment means, medical history, and medication in the form of node network graph. Such a centralized and clear structure can help researchers quickly clarify the relations and differences among numerous and complex knowledge points (16). AI mining engine is constructed to identify valuable hidden relations from the huge breast cancer-specific database and analyze such relations through clustering, attribute comparison and AI active learning, and the results are reviewed by experts and incorporated into the knowledge graph if passing the review. After deep knowledge data mining, the cross-departmental and even cross-hospital knowledge relations can be established only for dynamic knowledge graphs based on multivariate knowledge graph, thus expanding the entity set, relation set and triple set of knowledge graph. Meanwhile, the entity is not limited to the single representation only in breast cancer-related terms. The traditional knowledge graph is out of use, and the current knowledge graph integrates multi-modal knowledge, and displays, represents and utilizes medical data in various forms to the largest extent for the convenience of learning and understanding by researchers (17, 18). The partial knowledge graph constructed is shown in Figure 6.

## Maintenance of Data Security and Sharing of the Achievements in Disease-Specific Database

After the breast cancer-specific database system is constructed, in order to maximize its value in clinical practice and scientific research, rather than being limited to the inquiry and use in the Center and the Hospital, the data should be shared to multiple parties. However, medical data are particularly sensitive, so the following solutions will be adopted to share the data under the premise of ensuring the data security and patient privacy.

Due to the high security requirements of medical information data and the small volume of data in a single institution, federated learning, or multi-party secure computation can be considered to achieve joint use of data by multiple parties while ensuring data privacy and security. Essentially, both approaches limit the data use to the specified scope, which is effective to avoid data leakage and abuse. Federated learning is a distributed machine learning technique which collaborates the data modeling by multiple parties without data exchange (19). This model ensures the privacy of medical data and allows scientific research. Multi-party secure computation technically ensures that multiple parties of data cannot obtain the original data, and realizes collaborative computation without data leakage, that is, multiple parties run a computing task, machine learning task and data retrieval so as to obtain the final results based on common data, but data and intermediate calculation results will not be disclosed to any party in this process.

## Developing Data Value to Support Clinical Research

Support scientific research. The disease-specific database system will provide massive datasets for scientific research. Researchers can precisely filter target data according to their different research needs. Data analysis tools are also provided to improve data processing capabilities of the Hospital. Researchers can customize screening modes from the aspects of data sources, data time periods, data label types, verified knowledge, etc. to efficiently and accurately retrieve target data from massive data, thus indirectly improving clinical scientific research capabilities.

Support clinical practice. The diagnosis and treatment data of a breast cancer patient can be synchronized in real time through the “Breast Cancer-specific Database System” to form a data file which includes diagnostic imaging data, clinical pathology data, basic patient information, medical advice information, medication, surgery, radiotherapy, chemotherapy, cost settlement, etc., as well as the associated breast cancer knowledge database, etc. Combined with the “multi-modal breast cancer-specific knowledge graph” and based on the database-wide medical big data, various quantitative or qualitative big data machine learning algorithms are utilized for data analysis (20–22) to output the holographic knowledge portrait analysis reports of the patient's breast cancer risk profile, disease trend, clinical protocol, etc., such as the possibility of certain conclusion and the proportion of certain therapeutic regimen, providing the physicians with multi-dimensional and rich reference information, improving the ability of junior physicians in identification, diagnosis and treatment, and reducing the probability of missed diagnosis and misdiagnosis. Physicians can intuitively view, analyze and integrate multi-dimensional and multi-level holographic knowledge portrait, thus providing reference knowledge for accurate diagnosis and treatment of breast cancer based on the full-volume data. With the help of the auxiliary diagnostic system, physicians can provide more accurate therapeutic regimen based on the stage of the cancer and the patient's physical condition. Meanwhile, the intelligent auxiliary diagnosis system for breast cancer also provides a whole course management tool covering the examination, treatment and follow-up visit, so that the physicians can optimize the therapeutic regimen as appropriate in a timely manner, improve the treatment effect, and also provide more valuable data for the normalization and standardization of breast cancer treatment while using it (23).

Support teaching. Based on the whole-process therapeutic regimen in the breast cancer-specific database and the real physiological data of patients, theoretical learning and practice are carried out simultaneously for teachers and students. The most important thing is that the data are real and updated in real time update, so they are more instructive.

## Conclusion

A breast cancer-specific database system based on full data lifecycle, by integrating the data and processes of existing clinical data systems, accumulates knowledge database, provides standard access interface and back feeds business integration to promote the optimization and transformation of existing disease-specific research processes and form a closed loop for sustainable development. The disease-specific database system covers several disease-specific databases for conveniently saving and managing patient data in a systematic, standardized and accurate manner, so as to realize the tracking of breast cancer cases, and effectively develop teaching, scientific research and evaluation on the effects of various therapies for breast cancer. A scientific platform is created for research on breast cancer pathogenesis and etiology through comprehensive long-term longitudinal tracking and data comparison/analysis.

Clinical text analysis and knowledge extraction are conducted to integrate two heterogeneous clinical data sources, that is, unstructured medical record data and structured clinical data. New-generation information technologies, such as big data, NLP text parsing, data mining and knowledge graph, are deeply fused and applied to build a disease-specific database system based on full data lifecycle for the purpose of breast cancer disease characteristic analysis, so as to effectively develop teaching, scientific research and evaluation on the diagnosis and treatment of breast cancer and the follow-up visit tracking of cases, conduct comprehensive long-term longitudinal tracking and data comparison and analysis, and create a scientific platform for research on cancer pathogenesis and etiology. Big data and AI technology are utilized to provide continuous help for single disease of breast cancer before, during and after surgery, enable the physicians to deeply participate in the whole path of disease diagnosis and treatment, truly achieve accurate diagnosis and treatment planning, and break the data barriers between clinical departments. The governance and application of image data are emphasized to explore the image optimization algorithm and image recognition tool through database feedback and cyclic iteration optimization. The occurrence and development rules of relevant diseases are analyzed based on population categories to provide big data-based analysis and services for better clinical diagnosis and treatment, health management and clinical evidence-based medical research. Specialized research and disease-specific database are the focus of the connotation construction of the Hospital. The comprehensive hospitals in China can win a competitive advantage only by strengthening the construction of disciplines and also better meet the health service requirements of society and the country.

## Data Availability Statement

The datasets for this study are available from the corresponding author on reasonable request. Requests to access these datasets should be directed to yinjin@wchscu.cn.

## Ethics Statement

The studies involving human participants were reviewed and approved by the biomedical research ethics committee of West China Hospital (reference number: 20200427). Written informed consent for participation was not required for this study in accordance with the national legislation and the institutional requirements.

## Author Contributions

CX and YJ were responsible for the study's concept. YJ, WJ, JJ, and SY were responsible for data and project management. YJ and WJ performed the data cleaning and database construction. YJ, WJ, JJ, SY, QK, and CX drafted and revised the manuscript. All authors approved the final manuscript as submitted and agree to be accountable for all aspects of the work.

## Conflict of Interest

CX is employed by Chengdu Zhixin Electronic Technology Co., Ltd. The remaining authors declare that the research was conducted in the absence of any commercial or financial relationships that could be construed as a potential conflict of interest.
